# Intracellular symbiont *Symbiodolus* is vertically transmitted and widespread across insect orders

**DOI:** 10.1093/ismejo/wrae099

**Published:** 2024-06-14

**Authors:** Jürgen C Wierz, Philipp Dirksen, Roy Kirsch, Ronja Krüsemer, Benjamin Weiss, Yannick Pauchet, Tobias Engl, Martin Kaltenpoth

**Affiliations:** Department of Insect Symbiosis, Max Planck Institute for Chemical Ecology, 07745 Jena, Germany; Department of Insect Symbiosis, Max Planck Institute for Chemical Ecology, 07745 Jena, Germany; Department of Evolutionary Ecology, Institute of Organismic and Molecular Evolution, Johannes Gutenberg University, 55128 Mainz, Germany; Department of Insect Symbiosis, Max Planck Institute for Chemical Ecology, 07745 Jena, Germany; Department of Insect Symbiosis, Max Planck Institute for Chemical Ecology, 07745 Jena, Germany; Department of Insect Symbiosis, Max Planck Institute for Chemical Ecology, 07745 Jena, Germany; Department of Insect Symbiosis, Max Planck Institute for Chemical Ecology, 07745 Jena, Germany; Department of Insect Symbiosis, Max Planck Institute for Chemical Ecology, 07745 Jena, Germany; Department of Insect Symbiosis, Max Planck Institute for Chemical Ecology, 07745 Jena, Germany

**Keywords:** insect symbiont, vertical transmission, mixed mode transmission, transovarial, intracellular, secretion systems, genome erosion, parasitism-mutualism continuum

## Abstract

Insects engage in manifold interactions with bacteria that can shift along the parasitism–mutualism continuum. However, only a small number of bacterial taxa managed to successfully colonize a wide diversity of insects, by evolving mechanisms for host-cell entry, immune evasion, germline tropism, reproductive manipulation, and/or by providing benefits to the host that stabilize the symbiotic association. Here, we report on the discovery of an *Enterobacterales* endosymbiont (*Symbiodolus*, type species *Symbiodolus clandestinus*) that is widespread across at least six insect orders and occurs at high prevalence within host populations. Fluorescence *in situ* hybridization in several Coleopteran and one Dipteran species revealed *Symbiodolus*’ intracellular presence in all host life stages and across tissues, with a high abundance in female ovaries, indicating transovarial vertical transmission. Symbiont genome sequencing across 16 host taxa revealed a high degree of functional conservation in the eroding and transposon-rich genomes. All sequenced *Symbiodolus* genomes encode for multiple secretion systems, alongside effectors and toxin-antitoxin systems, which likely facilitate host-cell entry and interactions with the host. However, *Symbiodolus*-infected insects show no obvious signs of disease, and biosynthetic pathways for several amino acids and cofactors encoded by the bacterial genomes suggest that the symbionts may also be able to provide benefits to the hosts. A lack of host-symbiont cospeciation provides evidence for occasional horizontal transmission, so *Symbiodolus*’ success is likely based on a mixed transmission mode. Our findings uncover a hitherto undescribed and widespread insect endosymbiont that may present valuable opportunities to unravel the molecular underpinnings of symbiosis establishment and maintenance.

## Introduction

Bacteria can be valuable symbiotic partners for eukaryotes [1], opening up new ecological niches for their hosts by supplying limiting nutrients, detoxifying or digestive enzymes, and/or protective compounds. On the other hand, bacterial pathogens can cause disease and severely impair host fitness. In many cases, however, the impact of symbiotic microbes on host fitness is not clear, and host–microbe interactions often shift along the parasite–mutualist continuum [2]. Whereas numerous bacteria can opportunistically interact with a host, certain taxa are well adapted to an obligate symbiotic lifestyle [3, 4] and have evolved sophisticated mechanisms to establish and maintain symbiosis [5, 6].

Insects form the most speciose animal class on the planet, and their ecology is often tightly intertwined with interactions with bacteria. Whereas many bacteria are associated with only a few, closely related insect hosts [7], some others display remarkable adaptations for colonizing and inhabiting invertebrate cells and consequently exhibit an enormous distribution and abundance across insect orders. Although these specialized insect symbionts with broad host range are evolutionarily successful, they only comprise a comparatively small number of taxa belonging to the phyla *Bacteroidota* (e.g. *Cardinium*, as well as a large clade including *Blattabacterium*, *Karelsulcia*, and *Shikimatogenerans* [8–10]), *Mycoplasmatota* (e.g. *Spiroplasma* [11]), and *Pseudomonadota* (e.g. the *Alphaproteobacteria Rickettsia* and *Wolbachia* [12, 13], as well as the *Gammaproteobacteria Arsenophonus*, *Sodalis*, *Rickettsiella*, and a large group of symbionts including *Buchnera* and *Nardonella*, among others [14–17]). Common characteristics of these successful symbionts are an intracellular localization, sometimes with a broad tissue tropism, and specific mechanisms ensuring transmission.

There are three major strategies to become a specialized insect symbiont with broad host range and establish evolutionary stable associations with many different insect hosts, and all of the bacteria mentioned above utilize at least one of them. The first strategy (“parasite”) is to evolve mechanisms to infectiously colonize insects and inhabit host cells, often at the expense of the host. Pivotal for such antagonistic behavior is the ability to evade the host immune system, e.g. via modifications of the cell envelope [5, 18–20]. For host cell entry, bacteria utilize invasins/autotransporters or secretion systems to translocate effectors that mediate uptake [21, 22]. Adaptations that bypass host control facilitate horizontal transmission, and infection of the germline can allow for vertical transmission. For symbionts colonizing the host germline, the second strategy (“reproductive manipulator”) can be to manipulate host reproduction to the symbiont’s advantage, allowing for its rapid spread within a host population [13]. This is usually achieved by manipulating the host to produce more female offspring, the symbiont-transmitting sex, or by conferring an advantage to symbiont-infected vs. -uninfected females in crosses with infected males (i.e. cytoplasmic incompatibility) [23]. However, modeling predicts that reproductive manipulation alone cannot explain the success of bacteria like *Wolbachia*, so it is hypothesized that this strategy may be coupled with context-dependent fitness benefits to their hosts [24–26]. The third strategy (“beneficial symbiont”) is to provide fitness benefits to the host. In this type of interaction, host-level selection often ensures successful transmission and maintenance across host generations [27]. This scenario can lead to long periods of host-symbiont coevolution and co-diversification, resulting in large and diverse host and symbiont clades [28]. Usually, obligate symbionts are characterized by the localization in distinct host tissues (bacteriomes or other symbiotic organs), which may facilitate nutrient transport [29], avoid immune stimulation of the host [30], and/or allow for the control of symbiont proliferation by the host [31].

Although some of the specialized insect symbionts with broad host range follow one of the three strategies, combinations and transitions between strategies occur, with reported cases of both parasites and reproductive manipulators evolving into beneficial symbionts [32, 33]. Unfortunately, however, insights into the evolutionary transitions between parasitic and mutualistic associations are currently hampered by the lack of detailed functional data on many of the widespread symbiotic interactions, especially those involving bacteria that are commonly assumed to be parasites or reproductive manipulators. Additionally, the small number of insect-associated bacterial taxa in these two categories limits the potential for drawing generalizable conclusions on the mechanisms, fitness consequences, and evolutionary dynamics underlying the specialized insect-associated lifestyle.

Here, we describe the widespread occurrence of a clade of hitherto undescribed *Enterobacterales* symbionts that we identified across the six insect orders Coleoptera, Diptera, Ephemeroptera, Hemiptera, Lepidoptera, and Siphonaptera. We characterize the endosymbiont’s (ES) intracellular localization and tissue tropism across multiple host taxa, assess its prevalence in host populations, and provide functional insights based on genome sequences of the symbionts across 16 host taxa. We propose the new genus “*Symbiodolus*” for these bacteria in reference to the symbiotic lifestyle and the daimon of trickery, disguise, and deception from Greek and Roman mythology (Dolus), based on the long evasion of the symbiont from scientific investigation. As the symbiont likely also evades host immunity, we anticipate that future studies may provide a double meaning to the name. Furthermore, for one clade of very closely related strains, we propose the new species “*Symbiodolus clandestinus*.” We will use the genus name throughout the manuscript to refer to all strains investigated in this study, as they share a lot of characteristics. Nonetheless, future discoveries may reveal strains with different traits.

## Material and methods

### Sampling

Chrysomelidae, Curculionidae, and Silvanidae specimens were collected in and around Mainz and Jena, Germany. Specimens of *Pactopus hornii* (Throscidae) were acquired from the Canada Center for DNA Barcoding, and specimens of *Chironomus riparius* were obtained from two laboratory-reared populations that originate from Germany and Spain, respectively, and were maintained at the University of Frankfurt. Several sequences were obtained from NCBI, in particular the chromosome sequence of the ES of *Chironomus riparius* (GenBank OU907312) as well as the 16S rRNA gene sequences for the ESs of *Meligethes atratus* (GenBank SRR16308437), *Paracorethrura iocnemis* (GenBank OQ099617), and *Irenimus aequalis* (GenBank KJ494864). Information on symbionts from host taxa in the SRA was acquired after assembling the respective read libraries (Supplementary file 03).

### DNA extraction

Methods for DNA extraction varied between samples depending on purpose. For the analysis of symbiont prevalence and titer in *Oulema gallaeciana* and *Oulema melanopus*, whole beetles were individually extracted with the Quick DNA Tissue/Insect 96 Kit (Zymo Research, Irvine, CA, USA) following the manufacturer’s instructions. For the sequencing of the *Oulema gallaeciana* symbiont genome, DNA from individual beetles was extracted with the Nanobind Tissue Big DNA Kit (Circulomics, Baltimore, MD, USA) and the obtained DNA was subsequently used for Nanopore and Illumina sequencing. For all other analysis, including the 16S rRNA gene amplicon sequencing of Curculionidae, *Silvanoprus fagi*, and *P. hornii*, the Illumina shotgun sequencing of *Nedyus quadrimaculatus*, *Phyllobius maculicornis*, *Phyllobius roboretanus*, *Polydrusus formosus*, *S. fagi*, and *P. hornii*, as well as the Sanger sequencing of *Chironomus riparius*, the DNA was extracted with the Epicentre MasterPure Complete DNA and RNA Purification Kit (Epicentre, Illumina Inc., San Diego, CA, USA) according to the manufacturer’s instructions, including RNase digestion.

### Diagnostic and quantitative PCR

Diagnostic PCRs were performed with a Mastercycler EP Gradient S Thermocycler (Eppendorf AG, Hamburg, Germany), using a reaction mix containing 9.5 μL ultrapure H2O, 12.5 μL of Q5 High-Fidelity 2X Master Mix (NEB, Ipswich, MA, USA), 1 μL of both forward and reverse primer (each 10 pmol/μl), and 1 μL template. To identify the symbiont in *Chironomus riparius,* the 16S rRNA gene was amplified using the general primers fD1 and rP2 or the specific primer pair Chiro_ripa_ES_fwd01 and Chiro_ripa_ES_rev01 that was designed based on the available 16S rRNA gene sequence of the symbiont (Supplementary Table S1).

Quantitative PCRs (qPCRs) for symbiont titer measurements in *O*. *gallaeciana* and *O. melanopus* were performed on a CFX Connect Real-Time PCR Detection System (BIO-RAD, Hercules, CA, USA). The reaction cocktail was composed of 10 μl Biozym Blue S'Green (Biozym, Hessisch Oldendorf, Germany), 7.4 μl H_2_O, 0.8 μl each of forward primer Ogalla_fwd01 and reverse primer Ogalla_rev02 (each 10 pmol/μl), and 1 μl of 1 ng/μl template. For absolute quantification of symbiont 16S rRNA gene copy numbers, a standard curve created as a 10-fold dilution series of the corresponding purified PCR product was used, after measuring the concentration of the PCR product with a Qubit 4 Fluorometer (Invitrogen by Thermo Fisher Scientific, MA, USA).

### Sequencing

#### Sanger sequencing for symbiont confirmation

Following PCR, samples were purified with the Zymo Research DNA Clean & Concentrator-5 kit (Zymo Research, Irvine, CA, USA) following the manufacturer’s instructions. Sequencing was performed with a Hitachi 3730XL DNA Analyzer (Applied Biosystems by Thermo Fisher Scientific, MA, USA).

#### Microbial community profiling by 16S rRNA gene amplicon sequencing

High-throughput amplicon sequencing of bacterial 16S rRNA genes was done commercially (StarSeq, Mainz, Germany) on a MiSeq System (Illumina Inc., San Diego, CA, USA) using V3 reagents and 25% PhiX to balance base composition. Sequencing was performed in a paired-end approach with a read length of 300 nt, amplifying the V3-V4 region with primers 341f and 806bR (Supplementary Table S1). Amplicon sequence variants (ASVs) were identified based on the received reads after read trimming, quality filtering, dereplicating, and chimera removal in R utilizing the package DADA2 [34]. Taxonomy was assigned by using the pre-trained classifier Silva 138.1 [35, 36]. Prior to plotting, all reads identified as chloroplast or mitochondria were removed, and subsequently, all samples with less than 1000 reads were omitted.

#### Symbiont genome sequencing

The generation of Illumina short-read sequences for symbiont genome sequencing was done at the Max Planck-Genome Center (Cologne, Germany). A PCR-free DNA library was generated using the TruSeq DNA PCR-Free High Throughput Library Prep Kit (Illumina) and double-indexed adapter tags. Paired-end reads (2 × 250 bp) were generated by sequencing the library on a HiSeq 3000 System (Illumina Inc., San Diego, CA, USA) in Rapid Mode. For obtaining one high-quality complete genome of *Symbiodolus*, we obtained long Nanopore reads based on DNA from three individual *Oulema gallaeciana* beetles. Samples were treated with the Short Read Eliminator Kit XS (Circulomics, Baltimore, MD, USA) to selectively precipitate high molecular weight (HMW) fragments. Sequencing libraries were constructed per individual beetle using the HMW DNA as input for the Nanopore LSK-109 ligation kit (Oxford Nanopore Technologies, UK) following the manufacturer’s protocol. A total of 30.3 Gb were generated from R 9.4.1 MinION flow cells and bases were called by GUPPY v4.0.11 [37] with high-accuracy option (dna_r9.4.1_450bps_hac.cfg model).

### Genome assembly, annotation, and analysis

Genomes were assembled using Illumina reads only, with the exception of the *O*. *gallaeciana* symbiont (see below). For this, paired Illumina sequence reads were uploaded to KBase [38] and read quality was evaluated utilizing “FastQC v0.11.5-v0.11.9.” Afterward, reads were trimmed with “Trimmomatic v0.36” [39] and the trimmed reads were subsequently assembled with “metaSPAdes v3.13.0-v3.15.3” [40] and “MEGAHIT v1.2.9” [41].

The genome of the symbiont of *O*. *gallaeciana* was assembled using long reads from Nanopore sequencing, utilizing Flye v2.8.3 [42] with “—meta” option. The generated assembly was polished four times with Racon v1.4.13 [43] with (-m 8 -x -6 -g -8 -w 500) option and then further polished once with Medaka v1.0.3 (https://nanoporetech.github.io/medaka) with the r941_min_high_g344 model using the MinION raw reads. Subsequent polishing with Illumina short reads was performed using ntHits v0.1.1 (https://github.com/bcgsc/nthits) and ntEdit v1.3.2 [44] with the default settings. Duplications (heterozygous regions) were purged with PURGEhaplotigs v1.0.3 [45] and this ended up in the final genome assembly.

After assembly, (draft-) genomes were annotated in KBase using Prokka v1.14.5 [46]. In addition, analysis was performed with the aid of KEGG: Kyoto Encyclopedia of Genes and Genomes [47–49] and the InterPro database [50]. Synteny analyses were done with clinker [51] showing only the highest similarity links between genes. For the comparison, the assembled contigs of the draft genomes of the ES of *S. fagi* and ES of *Hystrichopsylla weida* were concatenated.

### SRA search

To study the prevalence of *Symbiodolus* symbionts within the Arthropoda, we used PhyloFlash v3.4 [52] to reconstruct full length small ribosomal subunit (SSU) sequences from whole genome sequencing projects stored in the NCBI Sequence Read Archive (SRA). First, we identified relevant data sets for Coleoptera and Arthropoda with the search queries “Coleoptera”[Organism] AND (“filetype fastq”[Properties] AND “strategy wgs”[Properties] AND “platform illumina”[Properties] AND “biomol dna”[Properties] AND “library layout paired”[Properties]) as well as “Arthropoda”[Organism] AND (“filetype fastq”[Properties] AND “strategy wgs”[Properties] AND “platform illumina”[Properties] AND “biomol dna”[Properties] AND “library layout paired”[Properties]), respectively. For computational feasibility, we limited the Arthropoda results to a single genome per genus, selecting the largest read archive if multiple were available, resulting in a final list of 3285 datasets. Second, each dataset was downloaded, its read length calculated with awk-scripting, and SSU sequences reconstructed with PhyloFlash. Finally, the obtained sequences were blasted and we selected SRA-stored libraries that contained a 16S rRNA gene sequence whose closest hit was to GenBank *OU907312* or GenBank *KJ494864* entries.

### Phylogenetic reconstruction

For some of the strains, a complete genome was not available, hence we used the 16S rRNA gene to understand the relationship of *Symbiodolus* within the Proteobacteria. We reconstructed a maximum likelihood-based phylogenetic tree of all aligned *Symbiodolus* 16S rRNA gene sequences using IQ-Tree (v2.2.2.3, [53]). The best model was "TPM3 + I + R4" as automatically determined by ModelFinder [54]. Tree search utilized the thorough nearest neighbor interchange (NNI) option (−allnni). Branch support was estimated using 10 000 ultrafast bootstraps [55] optimized via additional NNI based on bootstrap alignments (−bnni). To confirm the phylogenetic relationships, a phylogeny based on available (draft-) genomes was created with the help of KBase [38] utilizing “Insert Set of Genomes Into SpeciesTree - v2.2.0.” This aligned the sequences of 49 core universal marker genes defined by COG (Clusters of Orthologous Groups) gene families of user provided genomes with publicly available genomes of closely related bacteria and created a phylogenetic tree using an approximately-maximum-likelihood algorithm.

### Symbiont prevalence and titer

Field caught adults of *O*. *gallaeciana* and *O. melanopus* were kept in net cages (30 cm × 30 cm × 30 cm) at 24°C, 60% humidity with a 16/8 day/night cycle. A small tray (about 6 cm × 6 cm) of 7-day-old wheat plants was placed in the cage and once per week another plant was added. Each plant was left within the cage for 3 weeks. After 4 weeks, living beetles were collected and individually frozen until DNA extraction. Obtained DNA was used to measure symbiont prevalence and titer via qPCR.

### Determining the sex ratio of *O. gallaeciana* and *O. melanopus*

The sex of 46 *O*. *gallaeciana* and 80 *O. melanopus* field-caught adults (Jena, Germany) was determined by identifying the aedeagus of males by dissecting.

### Fluorescence *in situ* hybridization

For the localization of symbionts, we conducted fluorescence *in situ* hybridization (FISH) for adult specimens of *C. riparius*, *N. quadrimaculatus*, *O*. *gallaeciana*, and *P. hornii*, eggs of *O*. *gallaeciana*, and larvae of *C. riparius*, *O*. *gallaeciana*, and *O. melanopus*. Whole individuals of the different developmental stages and species were fixed in 4% PFA in 80% tertiary-butanol. After washing the samples in 80% tertiary-butanol for 4 times, they were dehydrated in a series of ascending concentration (90%, 96%, absolute) of tertiary-butanol, followed by three stages of acetone. Afterwards, they were embedded in Technovit 8100 (Heraeus Kulzer) according to the manufacturer’s instructions. With a glass knife, 8-μm-thick transversal or sagittal histological sections were cut on a Leica RM 2245 microtome and placed on microscope slides. To stain the bacteria, 100–150 μl hybridization mixture was applied to each slide, which were subsequently covered with a glass cover slip, and then hybridized over night at 50°C in a humid box. The hybridization mix consisted of hybridization buffer (0.9 M NaCl, 0.02 M Tris/HCl (pH = 8), 0.01% SDS), fluorescently labeled oligonucleotide probes with a concentration of 0.5 μM to mark bacteria and 0.5 mg/ml DAPI for host cell counterstaining. The probe EUB338 was used in all samples, targeting general bacteria (Supplementary Table S1). The *Pactopus* sample additionally used probe EUB784 for general bacteria staining. Probe Thros_Phorni_Entero_cy3 was used for samples containing *Symbiodolus* strains falling in clade 3 (i.e. *S. clandestinus*), labeling the specific symbiont. For samples of *C. riparius*, probe Chiro_ripa02_ES_cy3 was used instead to label *Symbiodolus*. To stain *Wolbachia* bacteria, probes Wolb_W2-Cy5 and Wolb_Wol3_Cy5 were additionally used in all samples except *C. riparius* and *P. hornii* samples. After hybridization, the glass cover slips were discarded, slides were submerged in wash buffer, and washed at 50°C for 2 hours, with an additional washing step in distilled water for 20 minutes. The wash buffer contained 0.1 M NaCl, 0.02 M Tris/HCl (pH = 8), 5 mM EDTA, and 0.01% SDS. Once washing was completed, 30 μl of VectaShield was applied to each slide and a glass cover slip sealed the sample. For visualization, samples were viewed under a Leica THUNDER imager DMi8 (Leica, Wetzlar, Germany) and the obtained images were processed in the Leica Application Suite X software (Leica, Wetzlar, Germany) with the small volume computational clearing algorithm.

## Results

### Symbiont distribution and phylogenetic affiliation

During microbiota profiling studies in Chrysomelidae and Curculionidae, we repeatedly came across 16S rRNA gene sequences that exhibited very high sequence similarity (>99%), and the only similar sequence found in the NCBI database originated from a bacterial community profiling study of the weevil *Irenimus aequalis* from New Zealand (GenBank: KJ494864). After systematically revisiting our available microbiota profiling datasets as well as the NCBI SRA archive, we discovered *Symbiodolus* in 23 distinct host species, spanning 13 families across the six insect orders Coleoptera, Diptera, Ephemeroptera, Hemiptera, Lepidoptera, and Siphonaptera (Supplementary Table S2).

**Figure 1:**
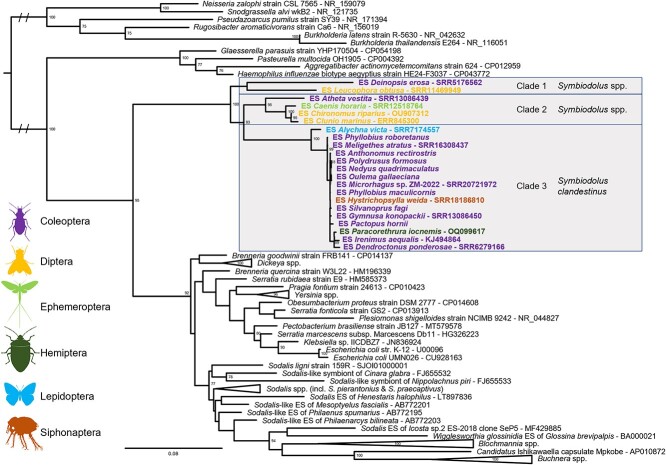
Phylogenetic reconstruction of *Symbiodolus* endosymbiont strains (ES) of various hosts with representative *Gammaproteobacteria* and an outgroup consisting of *Betaproteobacteria* based on aligned 16S rRNA gene sequences. The phylogeny was reconstructed using a maximum likelihood-based method using a "TPM3 + I + R4" model, and node labels indicate branch support as estimated by 10 000 ultrafast bootstraps optimized via additional NNI based on bootstrap alignments (only values above 70 are shown); all *Symbiodolus* formed a monophyletic clade with three subclades as highlighted, and taxa name colors specify host order as indicated on the left.

Based on 16S rRNA gene sequences, we reconstructed the phylogeny of the *Symbiodolus* symbionts (Fig. 1, Supplementary Fig. S1, see online supplementary material for a color version of this figure). The *Symbiodolus* strains formed a well-supported monophyletic clade within the *Gammaproteobacteria* distinct from all other known *Enterobacterales*. Within this monophyletic group, the sequences clustered into three separate clades. Even though the limited information of the 16S rRNA gene led to low support values for the branches within each *Symbiodolus* cluster, the three clades were also recovered with high support from a phylogeny based on available (draft-) genomes (Supplementary Fig. S2, see online supplementary material for a color version of this figure). Closest relatives were some *Brenneria*, *Serratia*, *Sodalis*, and *Yersinia* strains, each equally distant with a 16S rRNA gene sequence similarity of ~90%. Despite the large phylogenetic distance of their hosts, the different *Symbiodolus* strains showed remarkable similarity. This similarity was highest within clades, and the nucleotide identity of the 16S rRNA gene of strains in clades 1, 2, and 3 was 92.3%, 96.4%–99.4%, and 97.9%–100%, respectively, whereas between clades, the 16S rRNA gene sequence similarity ranged from 89.3%–95.1%.

### High symbiont prevalence in infected populations

We examined *Symbiodolus*’ prevalence in host populations in order to draw conclusions on its transmission success. Symbiont presence was assessed via diagnostic PCR for *C. riparius* (22/22 screened specimens harbored *Symbiodolus*), quantitative PCR (qPCR) for *O. gallaeciana* (23/23) and *O. melanopus* (20/20), and microbial community profiling for *Anthonomus rectirostris* (9/10) and *N. quadrimaculatus* (10/11). Thus, prevalence was consistently very high, with 90%–100% of individuals carrying *Symbiodolus* in all five species. Species with less than 10 screened individuals were not taken into consideration for the evaluation of symbiont presence.

**Figure 2:**
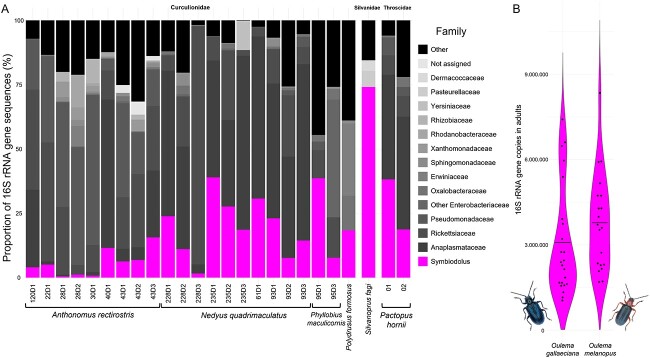
Relative and absolute abundance of *Symbiodolus* across different insect hosts; (A) bacterial community composition of various Curculionidae, *S. fagi* (Silvanidae), and *P. hornii* (Throscidae) beetles. Each bar depicts the relative abundance of bacterial ASVs within an individual beetle, identified at family level by DADA2 analysis of the 16S rRNA gene; *Symbiodolus* symbiont is highlighted in magenta, all other taxa are displayed in different shades of gray; (B) violin plot of the 16S rRNA gene copy number as a proxy for symbiont titer in adults of *O*. g*allaeciana* (left, *N* = 23) and *O. melanopus* (right, *N* = 20); black dots represent individual data points and horizontal bars represent the mean; beetle pictures from Wikimedia commons (U. Schmidt).

Analyzing the titers of bacterial symbionts in hosts can help to interpret their potential relevance in the system. Relative symbiont abundance determined via 16S rRNA gene amplicon sequencing varied greatly across individuals, ranging from 0.5% to 74% (Fig. 2a). Absolute symbiont titers, as measured in 16S rRNA gene copies by qPCR, were 3.09 ± 1.95*10^6^ (*N* = 23) copies within adult *O*. *gallaeciana* and 3.78 ± 1.72*10^6^ (*N* = 20) copies in adult *O. melanopus* (Fig. 2b). As each symbiont genome contains two 16S rRNA gene copies, these numbers translate to an average of 1.55*10^6^ and 1.89*10^6^ symbiont genome copies for *O*. *gallaeciana* and *O. melanopus*, respectively.

### Symbiont tissue tropism

**Figure 3:**
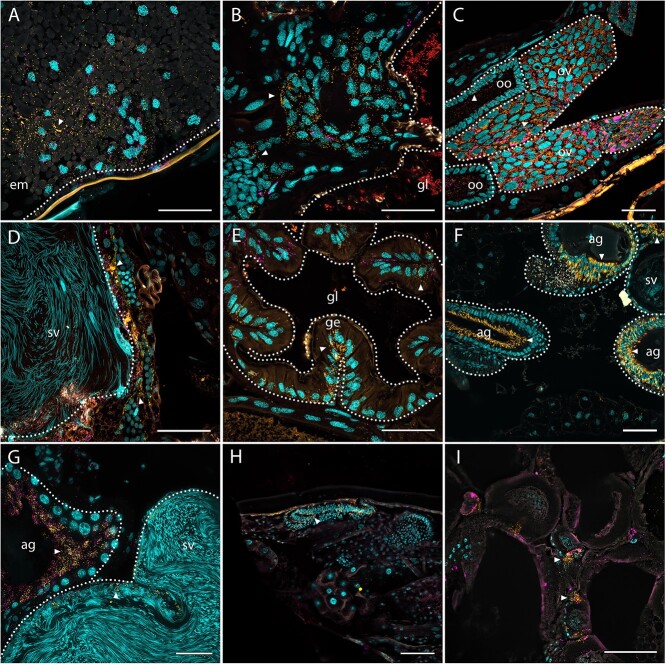
Localization of *Symbiodolus* in different host species and life stages; *S. clandestinus* was identified in *O*. *gallaeciana* (Chrysomelidae) eggs (A), larvae (B), adult females (C), and adult males (D), confirming the presence throughout all life stages via rRNA FISH; furthermore, the *Symbiodolus* symbiont was found in larvae of *O. melanopus* (Chrysomelidae) (E), adult males of *P. hornii* (Throscidae) (F), adult males of *N. quadrimaculatus* (Curculionidae) (G), larvae of *C. riparius* (Diptera, Chironomidae) (H), and female adults of *C. riparius* (I); *Symbiodolus* labeled in yellow were spread in all tissues (see arrowheads that highlight a few locations with *Symbiodolus*), most prominently in reproductive organs, and within these organs, it was co-localized with *Wolbachia*, labeled in magenta, in both *Ouelma* species as well as in *N. quadrimaculatus*; eubacterial staining is shown in red, host nuclei counterstaining in cyan, and autofluorescence in gray; used abbreviations are: embryo (em), gut lumen (gl), gut epithelium (ge), ovariole (ov), oocyte (oo), seminal vesicle (sv), and accessory gland (ag); bars = 50 μm.

Tissue localization of microbial symbionts within insect hosts can provide important information on their putative functional role and fitness impact on the host. We localized *Symbiodolus* in adults of *C. riparius*, *N. quadrimaculatus*, *O*. *gallaeciana*, and *P. hornii* via FISH. Across all four species, the symbionts were localized intracellularly in various tissues throughout the whole body, including fat body, muscles, and intestinal epithelium (Fig. 3). However, particularly high titers were observed in reproductive organs and tissues associated with them. Furthermore, symbionts were detected intracellularly in eggs of *O*. *gallaeciana* as well as in larvae of *C. riparius*, *O*. *gallaeciana*, and *O. melanopus*. The localization of *Symbiodolus* in the reproductive tissues as well as its presence across all life stages including eggs strongly suggests a vertical transmission route of the symbiont. *Symbiodolus* was consistently co-localized with *Wolbachia* in both *Oulema* species and in *N. quadrimaculatus*, whereas the presence of *Wolbachia* was not investigated in *C. riparius* and *P. hornii*.

### No sex ratio bias toward females

Based on tissue tropism, especially the high titers within reproductive organs, as well as the high prevalence within host populations, we speculated that *Symbiodolus* may be a reproductive manipulator. Therefore, we investigated the sex ratio of natural *O*. *gallaeciana* and *O. melanopus* populations by dissecting field-collected adult beetles. The results of 65.2% (30/46) males in *O*. *gallaeciana* and 62.5% (50/80) males in *O. melanopus* showed sex ratios that tended to be (*O*. *gallaeciana*: 1-sample proportions test with continuity correction; χ^2^ = 3.67, df = 1, *P* = .055) or were (*O. melanopus*: 1-sample proportions test with continuity correction; χ^2^ = 4.51, df = 1, *P* = .034) skewed toward males (Supplementary Fig. S3, see online supplementary material for a color version of this figure). Three out of four known mechanisms of symbiont-inflicted manipulation of the host population’s sex ratio result in a bias toward females: male killing, parthenogenesis induction, and feminization. The observed bias toward males in the two *Oulema* species indicates that the symbiont is probably not manipulating the sex ratio by any of these three mechanisms in these two host species. However, we cannot exclude the possibility that *Symbiodolus* is causing cytoplasmic incompatibility, which is not resulting in a biased sex ratio.

### Functional genome analysis of *Symbiodolus* symbionts

**Figure 4:**
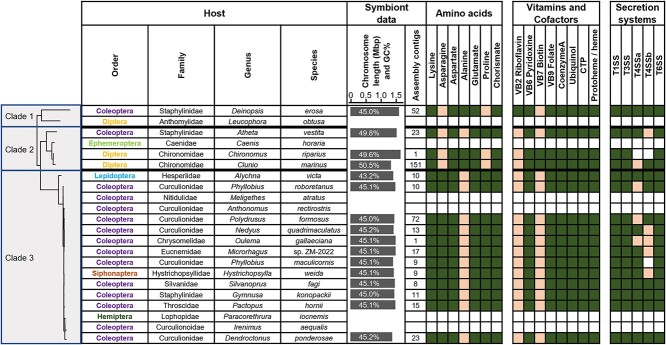
Genome characteristics and selected metabolic capabilities of different *Symbiodolus* strains; the phylogenetic tree on the left is taken from Fig. 1; host columns give the order, family, genus, and species of insect hosts for each analyzed symbiont strain; symbiont (draft-) genome lengths are depicted by bars, scale is in Mbp, and the largest genome was found in the ES of *C. riparius* with ~1.66 Mbp, the smallest was the incomplete assembly of the ES of *C. marinus* with ~1.35 Mbp; numbers inside bars are the respective GC contents (in %); the heatmap gives predicted functionality of amino acids pathways, vitamin and cofactors pathways, and secretion system machineries based on genomic information, and dark green fields indicate predicted functionality, light red fields indicate absence or non-functionality, and empty fields are missing data; for *C. riparius*, no definitive statement could be made about the presence or absence of the T4SS, as these genes were often found on plasmids and the available data did not include plasmids; for *P. maculicornis*, the presence of the T4SSb could not be conclusively confirmed nor disproved.

To elucidate the functional potential of *Symbiodolus* and gain insights into the possible interactions with its hosts, we sequenced and functionally characterized (draft-) genomes of *Symbiodolus* strains from 16 different host species (Fig. 4). Chromosome sizes ranged from about 1.4 to 1.6 Mbp. The short chromosome belonging to the ES of *C. marinus* was the most fragmented, so genome size is likely underestimated. Genomes of all *Symbiodolus* strains showed signs of erosion compared with related free-living bacteria (Fig. 4), consistent with a specialized symbiotic lifestyle. Although the glycolysis pathway seemed complete, several enzymes of the pentose phosphate pathway were not encoded and it was streamlined to only synthesize necessary precursors, e.g. for vitamin B6 (pyridoxine). The citrate cycle (TCA cycle) was incomplete with several steps missing. Still, all strains encoded the necessary genes for ATP synthase, NADPH production, and the cell envelope components peptidoglycan and cardiolipin. While it is possible that individual genes are missing from the assemblies, these patterns were consistent across all *Symbiodolus* (draft) genomes, making false negatives unlikely. There was a high number of genes annotated as transposases in the genomes of clade 1 *Symbiodolus* symbiont of *Deinopsis erosa* (i.e., 74), clade 2 *Symbiodolus* symbiont of *Chironomus riparius* (i.e., 96), and clade 3 *Symbiodolus* clandestinus symbiont of *Oulema gallaeciana* (i.e., 54). Other more fragmented draft genomes showed lower numbers of transposable elements. However, this may be an artifact, as these elements share high sequence similarity and therefore frequently interrupted contig assembly in Illumina short read assemblies, in turn causing fewer annotated transposase genes. The influence of transposases was also apparent in synteny analyses between different genomes, as even the closely related *S. clandestinus* strains in clade 3 showed several rearrangements of large blocks of the chromosome (Supplementary Fig. S4, see online supplementary material for a color version of this figure). A comparison of strains between clades showed numerous rearrangements and overall low levels of synteny (Supplementary Fig. S4, see online supplementary material for a color version of this figure).

#### Secretion systems, effectors, and toxins

To gain insights into possible molecular factors for host cell entry and injection of effectors, we screened the genomes for the presence of interaction machineries. Even though many assemblies only reached draft genome status, we identified the secretion systems type one (T1SS), three (T3SS), and six (T6SS) in every analyzed *Symbiodolus* genome (Fig. 4). For the T1SS, all three structural components were encoded: an ATP-binding cassette (ABC) transporter, a Membrane Fusion Protein (MFP), and an Outer Membrane Factor (OMF), together with several toxin-antitoxin genes. Furthermore, we discovered up to 17 genes potentially encoding the T3SS machinery. In addition to the translocators *SctA*, *SctB*, and *SctE*, we identified a well-known T3SS effector, encoded by the intimin gene, together with its translocated intimin receptor gene (*tir*), in all genomes of the analyzed *Symbiodolus*. However, the intimin gene appeared to be pseudogenized in the *Symbiodolus* strain of *C. riparius*, as the annotated gene region was only one third in length and lacked the passenger domain. The T6SS has 13 essential and conserved genes [56], named *TssA*-*TssM*. We identified all of them alongside the effectors *Hcp* and *VgrG* [57]. Another potential T6SS effector that we found was phospholipase A encoded by the gene *PldA* [58].

In contrast to the omnipresent T1SS, T3SS, and T6SS machineries, we found genes encoding the type four secretion system (T4SS) machinery in only some *Symbiodolus* genomes. The T4SS exists in various forms [59], and we detected T4SSa and/or T4SSb in several but not all symbiont genomes (Fig. 4). No clear pattern was observed between phylogenetic clade affiliation and presence/absence of the T4SS due to its patchy distribution. Moreover, the T4SS machinery genes were often found on contigs with higher assembly coverage and in close sequence proximity to the genes *parA* and *parB*, which encode chromosome partitioning proteins, as well as the plasmid replication initiation gene *repA*. It is therefore likely that the T4SS genes are located on a plasmid rather than on the chromosome. As plasmids are easier to be missed during metagenome assemblies, it is possible that the T4SS genes may have been missed in at least some of the *Symbiodolus* strains. However, we also re-mapped the raw reads of the metagenomes lacking T4SS genes to the plasmid sequences of *Symbiodolus* strains containing them and found no matches, indicating that some *Symbiodolus* strains indeed lack T4SS.

On top of these interaction machineries, *Symbiodolus* encoded a variety of toxin–antitoxin (TA) systems. These systems can be involved in normal physiology of bacteria as well as bacterial pathogenicity [60]. Among the detected TA systems were *fitB*/*fitA*, *mazF*/*mazE*, *higB*/*higA*, *ctpA*/*ctpB*, *vapC-1*/*vapB-1*, *yeoB*/*yefM*, and *yafQ*/*dinJ*, with no clear observed pattern of phylogenetic distribution (Supplementary file 02). We also scrutinized the genomes for genes that may be involved in reproductive manipulation of the insect hosts, but we did not detect factors such as *cifA*/*cifB* that can cause cytoplasmic incompatibility (CI), nor any other known genes responsible for reproductive manipulation [61–63].

#### Amino acid and cofactor metabolism

Besides secretion systems, *Symbiodolus* encoded pathways for the biosynthesis of several amino acids and cofactors (Fig. 4). These metabolites may be delivered to their hosts, thereby potentially providing a benefit. All analyzed strains are likely able to synthesize the amino acids aspartate, glutamate, lysine, and the aromatic amino acid precursor chorismate. There were minor differences between the strains from different clades. *Symbiodolus* symbionts from clade 1 as well as symbionts from Dipteran hosts in clade 2 could also synthesize alanine, and *S. clandestinus* symbionts from clade 3 were capable of synthetizing asparagine and proline. Beyond amino acids, all analyzed symbionts encoded the pathways for the cofactors coenzyme A, coenzyme Q precursor ubiquinol, cytidine triphosphate, heme, as well as the vitamins B6 (pyridoxine) and B9 (folate). Additionally, symbionts from clade 1 encoded the pathway for vitamin B2 (riboflavin) and symbionts from Dipteran hosts in clade 2 encoded the vitamin B7 (biotin) pathway (Fig. 4). As most genomes were not closed and therefore potentially incomplete, individual genes may have been missed.

#### Comparison to Sodalis praecaptivus

The genus *Sodalis* comprises taxa that range from free-living to obligately associated with an insect host [27], with *S. praecaptivus* being an environmental bacterium that is able to colonize insect tissues and cells [64, 65], thus providing an interesting comparison to *Symbiodolus*. A comparison of the genome content of *Symbiodolus* with *S. praecaptivus* revealed possible adaptations of *Symbiodolus* to a lifestyle inside insect hosts. *S. praecaptivus* retained many more capabilities, including a complete TCA cycle, a more extensive pentose phosphate pathway, biosynthetic pathways for all amino acids, and for several additional cofactors (e.g. thiamine (VB1), nicotinate (VB3), pantothenate (VB5), and biotin (VB7)). These capabilities were likely lost in *Symbiodolus*, as it probably obtains these metabolites from the host. In contrast, although *Symbiodolus* and *S. praecaptivus* share the presence of a T3SS, only *Symbiodolus* additionally encodes T4SS and T6SS, suggesting extended capabilities to interact with the insect host and with other bacteria.

## Discussion

We discovered a hitherto undescribed and widespread clade of bacterial symbionts that infects insects across at least six different orders. This wide distribution indicates that *Symbiodolus* is very adept at invading and colonizing various insect hosts, and it shows a high prevalence within host populations. FISH reveals an intracellular localization and broad tissue tropism across life stages, with a particular enrichment in adults’ reproductive tissues, consistent with a vertical transmission route. Functional genomic analyses reveal the presence of molecular machineries for host cell entry and the delivery of effectors, but also the presence of amino acid and vitamin biosynthesis pathways that could provide benefits to the host.

The broad phylogenetic distribution of *Symbiodolus* is astonishing, as such a widespread occurrence is only found in a small number of insect-associated bacteria (Fig. 1). These specialized insect symbionts with broad host range utilize different strategies to infect, persist in, and spread between their insect hosts. Some bacteria like *Sodalis* can invade host tissues and seem capable of horizontal and vertical transmission [21, 66]. Another strategy is the reproductive manipulation of the host, e.g. used by *Wolbachia*, to secure its prevalence in a population [13, 67]. However, even some host-beneficial bacteria can be found across many different host taxa, as seen, e.g. in *Karelsulcia muelleri* [28]. The spread of these symbionts may have occurred in the early stages of symbiosis, and they were further passed on later with host speciation.

A potential route for *Symbiodolus'* evolutionary success is its ability to invade host cells, which is reflected in the symbiont’s broad tissue tropism, including the germline (Fig. 3). This ability might be facilitated by the symbiont’s broad arsenal of systems putatively involved in the interaction with the host or with other microbes. Besides the universal SecYEG translocon, *Symbiodolus* encodes for T1SS, T3SS, and T6SS (Fig. 4). Furthermore, some strains also seem to carry plasmid-encoded T4SSa and/or T4SSb. Via the T1SS, bacteria can secrete small molecules such as toxins or antibiotics with various functions [68]. The T3SS functions as an injectisome that can inject effectors across both the inner and outer bacterial membranes into eukaryotic cells [68]. It is known to enable the invasion of eukaryotic cells, e.g. in the endosymbiont *Sodalis* associated with *Sitophilus* weevils [21]. T4SSs encompass a group of secretion machineries that inject macromolecules from Gram-negative bacteria into eukaryotic cells or other bacteria [69]. These can either mediate genetic exchange or deliver effectors to target cells. The T6SS is known for its wide variety of potential interactions with eukaryotes and bacteria, which can be pathogenic, commensalistic, or mutualistic, by translocating effectors and toxins [70–72]. We only identified a few effectors for the different secretion systems, but these indicate that the secretion machineries are likely used for host cell invasion: The T3SS associated intimin-tir operon allows parasitic bacteria to invade host cells [73]. Moreover, the T6SS associated phospholipase A (PldA) was shown to facilitate invasion of eukaryotic cells [58]. Reproductive manipulators such as *Cardinium*, *Spiroplasma*, and *Wolbachia* also utilize secretion systems, often T4SS [25, 74, 75], but their repertoire of secretion systems is usually smaller than that of *Symbiodolus*. Moreover, obligate beneficial symbionts usually do not retain any secretion systems. In addition to the aforementioned effectors, several of the identified TA systems (*fitB*/*fitA*, *mazF*/*mazE*, *vapC-1*/*vapB-1*, *yeoB*/*yefM*, and *yafQ*/*dinJ*) could play a role in interactions with the host. Among the potential functions are helping and speeding up the colonization of host tissues, aiding in intracellular survival and growth, promoting biofilm formation, and inducing necrosis of host cells [60, 76–78]. For example, *Rickettsia* bacteria seem to utilize *vapC* for the maintenance of the bacterium in its arthropod host, and a release of the toxin to a host cell can cause cell death [79, 80]. It is possible that along with these invasive capabilities, *Symbiodolus* is also able to evade the host immune system, but concrete evidence for this is still lacking.

Localization of *Symbiodolus* in *O*. *gallaeciana* via FISH revealed its presence in eggs, larvae, and adults (Fig. 3). Coupled with the observation that the symbiont is very abundant in the reproductive tissues across multiple host species, a transovarial transmission is highly likely. Concordantly, the high prevalence in multiple host species supports a high fidelity of vertical transmission. However, the occurrence of very similar *Symbiodolus* strains in phylogenetically distant host taxa indicates that horizontal transmission also occurs, at least occasionally. Some other unculturable endosymbionts with eroding or eroded genomes have been found to survive outside of the host for some time [81, 82], allowing for horizontal transmission. One example is the spread through shared food plants, a path that the insect symbionts *Rickettsia* in whiteflies and *Burkholderia* in Lagriinae beetles can use to transfer between individuals [82, 83]. Another possible vector are parasitoids, which have been experimentally shown to aid *Wolbachia*‘s spread within and between species [84, 85]. Although the mechanisms of horizontal transmission are still to be uncovered for *Symbiodolus*, its ability to be transmitted vertically and horizontally is reminiscent of many other facultative insect symbionts and has likely contributed to its evolutionary success [86, 87].

Another strategy for symbionts to be evolutionarily successful is the manipulation of their hosts’ reproduction to spread within host populations [88]. There are four main mechanisms of reproductive manipulation: feminization (FM), parthenogenesis induction (PI), embryonic male killing (MK), and cytoplasmic incompatibility (CI). Even though they have different implications for the host, all four increase the prevalence of the reproductive manipulator in female hosts (the transmitting sex) of the next generation, resulting in the symbiont’s spread within the host population [13, 89]. Influence of FM, PI, and MK lead to female biased sex ratios, whereas CI does not. The high *Symbiodolus* prevalence in *C. riparius*, *A. rectirostris*, *N. quadrimaculatus*, *O*. *gallaecia*, and *O. melanopus* could indicate that infected individuals produce more female offspring than non-infected ones, aiding the spread of the symbiont within the host population. However, the sex ratios in the two *Oulema* species showed no skew toward females, making FM, PI, and MK unlikely, at least in *Oulema* species. Furthermore, our genomic analysis did not reveal any obvious candidate genes involved in reproductive manipulation, including CI genes. However, the genetic basis of the symbionts’ ability to manipulate host reproduction can vary and remains unknown for most symbionts [90], so we cannot exclude the possibility that as yet unknown CI genes exist in the *Symbiodolus* genome*.*

Finally, *Symbiodolus* might be a beneficial symbiont for the insect hosts. The absence of obvious signs of disease in infected beetles indicates that the *Symbiodolus* symbiont is benign. Based on the diffuse localization of *Symbiodolus*, however, it is unlikely that is an obligate mutualistic symbiont. Furthermore, the high number of genes annotated as transposases suggests a more recent association at an intermediate stage of symbiosis, contrary to ancient beneficial symbionts which have a much-reduced amount of said genes [27]. Still, the compositions of *Symbiodolus*’ genomes indicate that the symbionts are capable of synthesizing various amino acids and cofactors that might be supplied to the host (Fig. 4). Even though nutritional supplementation is more common in bacteriome- or gut-localized symbionts, this is not a prerequisite, and even bacteria without any specialized localization and/or that are commonly considered parasitic, such as *Wolbachia*, can improve host fitness in a context-dependent manner by providing nutritional supplementation [26] or protection against pathogens [91]. Given the vertical transmission route, *Symbiodolus* could benefit from increasing host fitness, thereby increasing the number of offspring it can infect.

Among metabolites potentially provided by *Symbiodolus* is the essential amino acid lysine, a lack of which can severely impair insect fitness [92, 93]. Furthermore, *Symbiodolus* encodes the full shikimate pathway up until chorismate. This is a precursor for the aromatic amino acids phenylalanine and tyrosine, with the latter being a key metabolite for the biosynthesis, sclerotization, and melanization of the insect cuticle [94–96]. A deficiency in tyrosine can subsequently lead to the formation of a thinner, softer cuticle that is less able to protect the insect against biotic and abiotic stresses [11, 97, 98]. Concordantly, many insect taxa, and particularly various families of beetles, have recently been found to harbor obligate symbionts that supply their hosts with tyrosine precursors and thereby enhance cuticle biosynthesis [97–102]. Additionally, the symbiont uses chorismate as a precursor for ubiquinol synthesis, which in turn is required for the production of ATP by oxidative phosphorylation, as well as for vitamin B9 (folate) biosynthesis. In addition to amino acids, several cofactors might be provided to the host, particularly B-vitamins including B2, B6, B7, and B9 (Fig. 4). Vitamin B2 (riboflavin) functions as a precursor of flavin mononucleotide (FMN) and adenine dinucleotide (FAD) which are cofactors for flavoproteins and flavoenzymes. For insects, riboflavin can be crucial both during development and for adult survival [103]. The vitamers of B6 (pyridoxine) are involved in a wide variety of enzymatic activities. Symbiont-supplied B7 (biotin) can be crucial for the development, adult survival, and fecundity in various insects, and vitamin B9 (folate) is pivotal for the metabolism of amino acids and nucleic acids [103]. Hence, *Symbiodolus* has the genomic potential to provide nutritional supplements to the host that might be important for development and reproduction. The minor differences in potential supplementations between the *Symbiodolus* strains, e.g. between clade 2 symbionts of dipteran hosts and clade 3 symbionts (Fig. 4), might be explained by distinct nutritional needs of their respective hosts stemming from species-specific diets. However, no clear correlation between *Symbiodolus’* metabolic capabilities and host nutritional ecologies emerges based on the reported associations, and *Symbiodolus* appears to be associated with insects covering a broad ecological diversity, including herbivores, omnivores, and blood-feeders. Besides metabolic supplementation, other fitness enhancing contributions might be provided by the symbiont. In addition to the potentially antagonistic interactions facilitated by the secretion systems mentioned above, more mutualistic interactions with the host are possible [73]. Still, so far, it is unclear whether the symbiont provides any metabolites or other benefits to its hosts and context-dependent fitness benefits are especially difficult to predict from genomic data alone.

### Description of *Symbiodolus*—a new symbiont genus from various insect hosts

Monophyletic clade of intracellular symbionts within the *Gammaproteobacteria*; *Enterobacterales*, defined by its 16S rRNA gene sequences as well as (draft) genomes of 16 symbiont strains associated with insects across six different orders. For this so far uncultured, rod-shaped bacterium with an average length of about 1 μm, we propose the genus name ‘*Symbiodolus*’ ([Sym.bi.o.do'lus], **N.L. masc. n.**) for all strains in the monophyletic clade. This compound name implies a symbiotic association, but in a deceitful way, consisting of the terms “Symbio-” (**Gr. masc. /fem. n.***symbios*, companion) and “-dolus” (**L. masc. n.***dolus*, deceit, malice, deception, also the Roman and Greek daimon that is the personification of deception and fraud). Additionally, we propose the species name ‘*clandestinus*’ ([clan.de.sti'nus], **L. masc. Adj.***clandestinus*, secret or hidden) for *Symbiodolus* species of clade 3 (Fig. 1). The term *clandestinus* refers to the symbiont’s until now undescribed nature despite its wide distribution. Consequently, *Symbiodolus* species of clade 1 and 2 could be called *Symbiodolus* spp., and the host species affiliation of all strains can be indicated by strain names using a four-letter code, consisting of the first letter of the host genus name and the first three letters of the host species epithet. We deem this name fitting not only because of its apparent ability to invade host tissues but also because the symbiont has long eluded scientific discovery. The name *Symbiodolus cladestinus* has been endorsed by SeqCode Registry under the register list seqco.de/r:ysrrov43.

## Conclusion and outlook

Here, we describe an *Enterobacterales* symbiont present across at least six insect orders. Its phylogenetic distribution, intracellular localization, and broad tissue tropism indicate a mixed mode of transmission and the ability to colonize and spread between host cells, which is supported by the presence of genes encoding diverse secretion systems and effectors in the symbiont genome. Despite these putative virulence factors, *Symbiodolus* appears to be rather benign for host fitness and even has the genomic potential to provide fitness benefits to the host by supplementing limiting amino acids and B-vitamins. Many open questions about this symbiont remain. The distribution among insects alongside the age of the discovered symbiotic interactions is yet to determined, as is its fitness impact on the hosts. *Symbiodolus* may offer valuable opportunities to deepen our understanding of host-symbiont interactions. Given the comparatively large genome size (as compared to many other obligate intracellular symbionts of insects), the symbiont may be culturable and thus provide a new and tractable system to study intracellular symbioses, akin to *Sodalis* that has been recently used to establish a tractable symbiosis [65]. Deciphering *Symbiodolus*’ molecular tools used for host immune evasion, cell invasion, and vertical transmission, and comparing its mechanisms with a broader range of bacterial taxa may yield general insights on how bacteria become intracellular and establish persistent symbioses in insects.

## Supplementary Material

Wierz_et_al_Supplement_3rd_revision_changes_accepted_wrae099

Wierz_et_al_Supplement_02-ToxinAntitoxin_wrae099

Wierz_et_al_Supplement_03-all_SSU_in_SRA_wrae099

## Data Availability

The datasets presented in this study are available in online repositories. The sequences on which the microbial community analysis as well as the genome assemblies are based were stored on NCBI either under SRP488215 as part of the BioProject PRJNA1072544, or under SRP482428 as part of the BioProject PRJNA1062330 for the Throscidae samples. All symbiont genomes, the sequence alignment underlying the *Symbiodolus* phylogeny as well as an unfiltered output table from the microbial community analysis are available in Edmond, which is a research data repository for Max Planck researchers (https://doi.org/10.17617/3.NY3Y1R).
