# Supplementary material for: Intracellular symbiont Symbiodolus is vertically transmitted and widespread across insect orders
Source: ISME J. 2024 Jun 14;18(1):wrae099. doi: 10.1093/ismejo/wrae099 (PMC11322605; doi:10.1093/ismejo/wrae099)
Supplement: Wierz_et_al_Supplement_3rd_revision_changes_accepted_wrae099 [file wierz_et_al_supplement_3rd_revision_changes_accepted_wrae099.docx]

# **Supplementary material**

**Intracellular symbiont *Symbiodolus* is vertically transmitted and widespread across insect orders**

Jürgen C. Wierz^1^, Philipp Dirksen^1,2^, Roy Kirsch^1^, Ronja Krüsemer^1^, Benjamin Weiss^1^, Yannick Pauchet^1^, Tobias Engl^1^, Martin Kaltenpoth^1,^*

^1^ Department of Insect Symbiosis, Max Planck Institute for Chemical Ecology, Jena, Germany

^2^ Department of Evolutionary Ecology, Institute of Organismic and Molecular Evolution, Johannes Gutenberg University, Mainz, Germany

* Corresponding author: Martin Kaltenpoth, Department of Insect Symbiosis, Max Planck Institute for Chemical Ecology, Hans-Knöll-Str. 8, D-07745 Jena Germany, kaltenpoth@ice.mpg.de


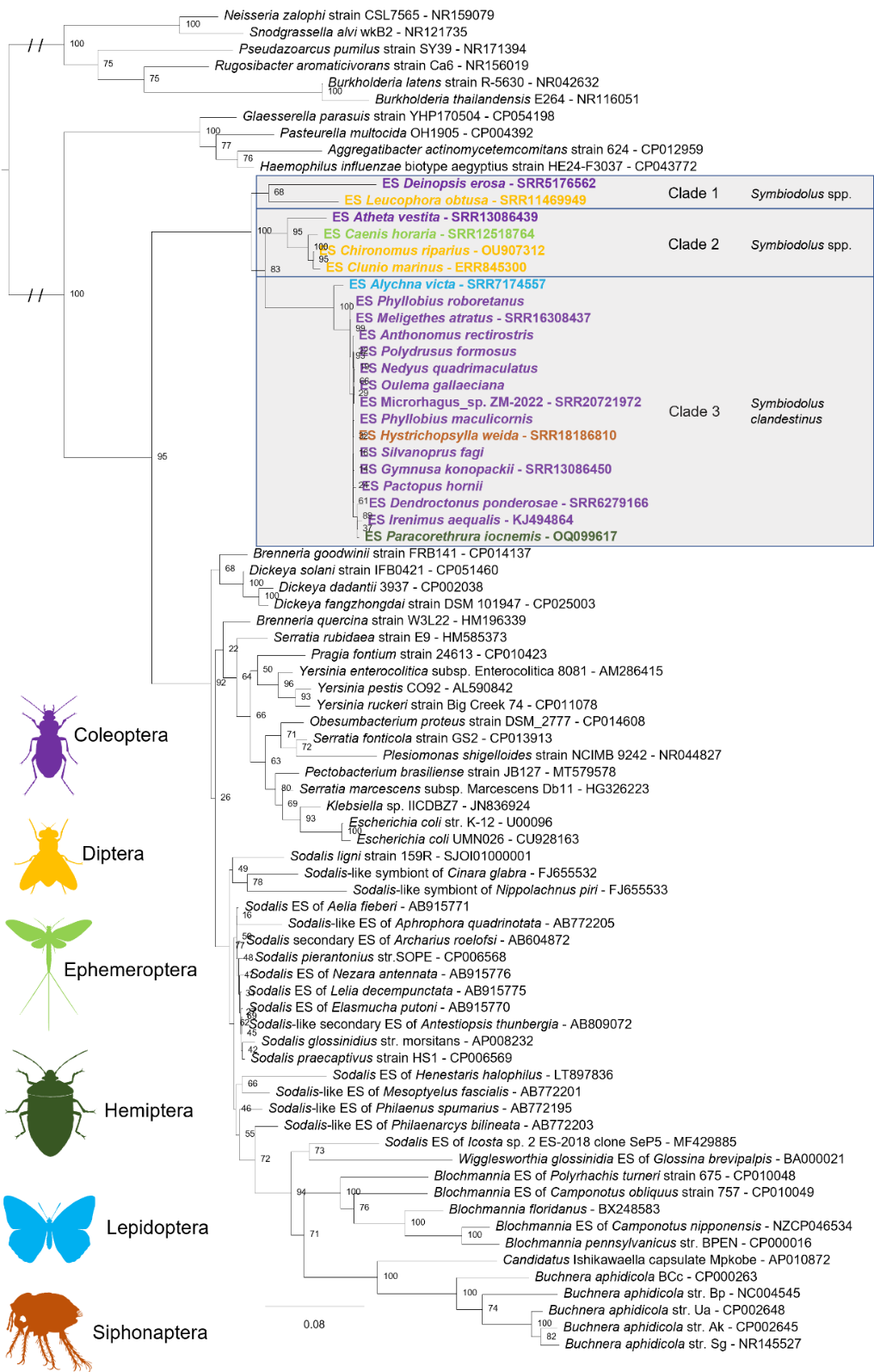


**Figure S1: Phylogenetic placement of *Symbiodolus* strains based on 16S rRNA gene sequence.**

Same as figure 2 but with non-collapsed clades. Phylogenetic reconstruction of *Symbiodolus* endosymbiont (ES) strains of various hosts within other representative *γ-Proteobacteria* and an outgroup consisting of *β-Proteobacteria* based on aligned 16S rRNA gene sequences. Tree was built with maximum likelihood-based method using a "TPM3+I+R4" model, node labels indicate branch support as estimated by 10,000 ultrafast bootstraps optimized via additional NNI based on bootstrap alignments. All *Symbiodolus* formed a monophyletic clade with three subclades as highlighted. Taxa name colors specify host order as indicated on the left, and host taxa association did not predict symbiont phylogenetic clade placement.


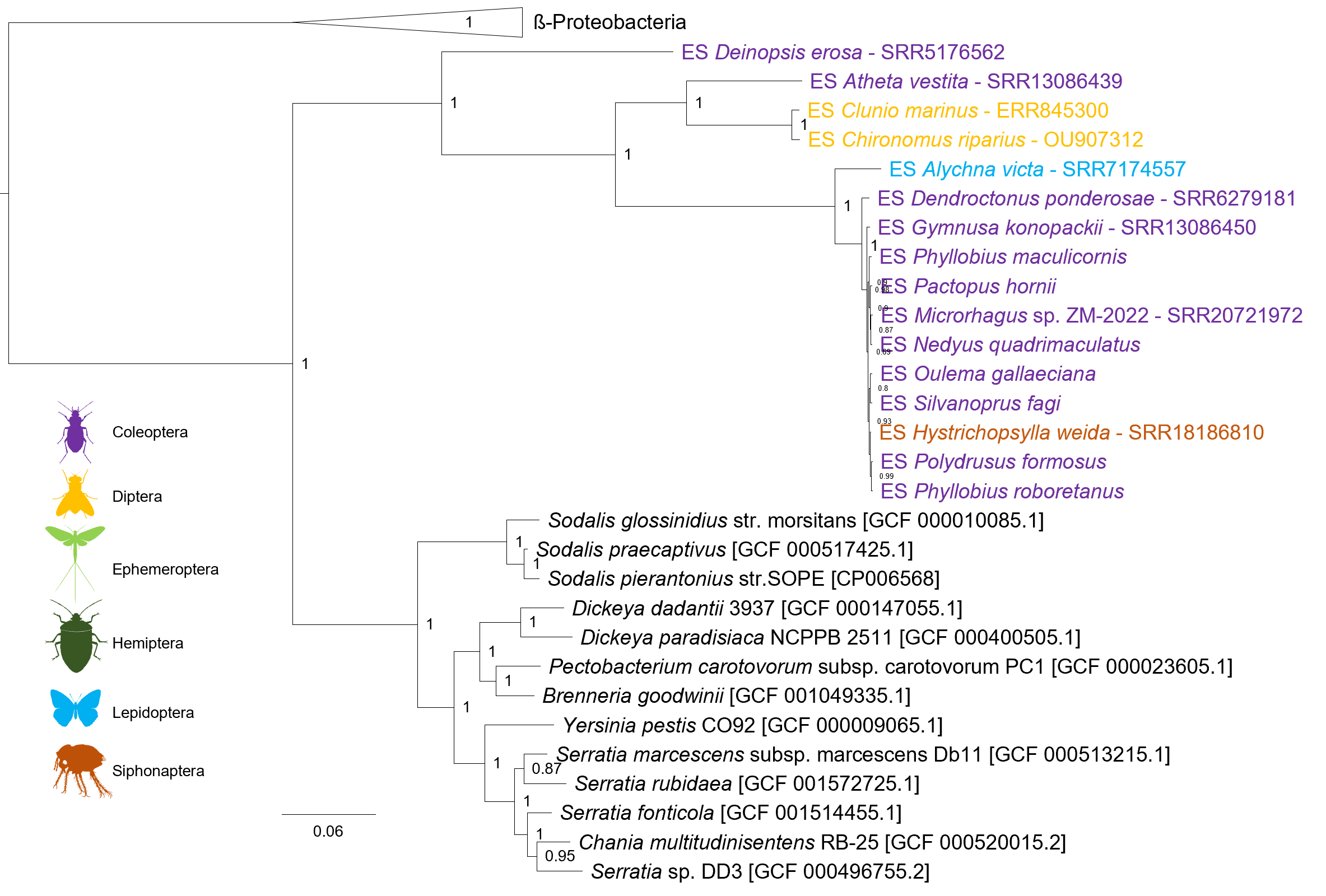


**Figure S2: Phylogenetic placement of *Symbiodolus* strains based on full genomes.**

Phylogenomic reconstruction illustrates the placement of *Symbiodolus* endosymbiont (ES) strains of various hosts based on a nucleotide alignment for a subset of 49 COGs. The tree was reconstructed using an approximately-maximum-likelihood algorithm (FastTree2), and node labels show local support values. The outgroup consists of *ß-Proteobacteria*.


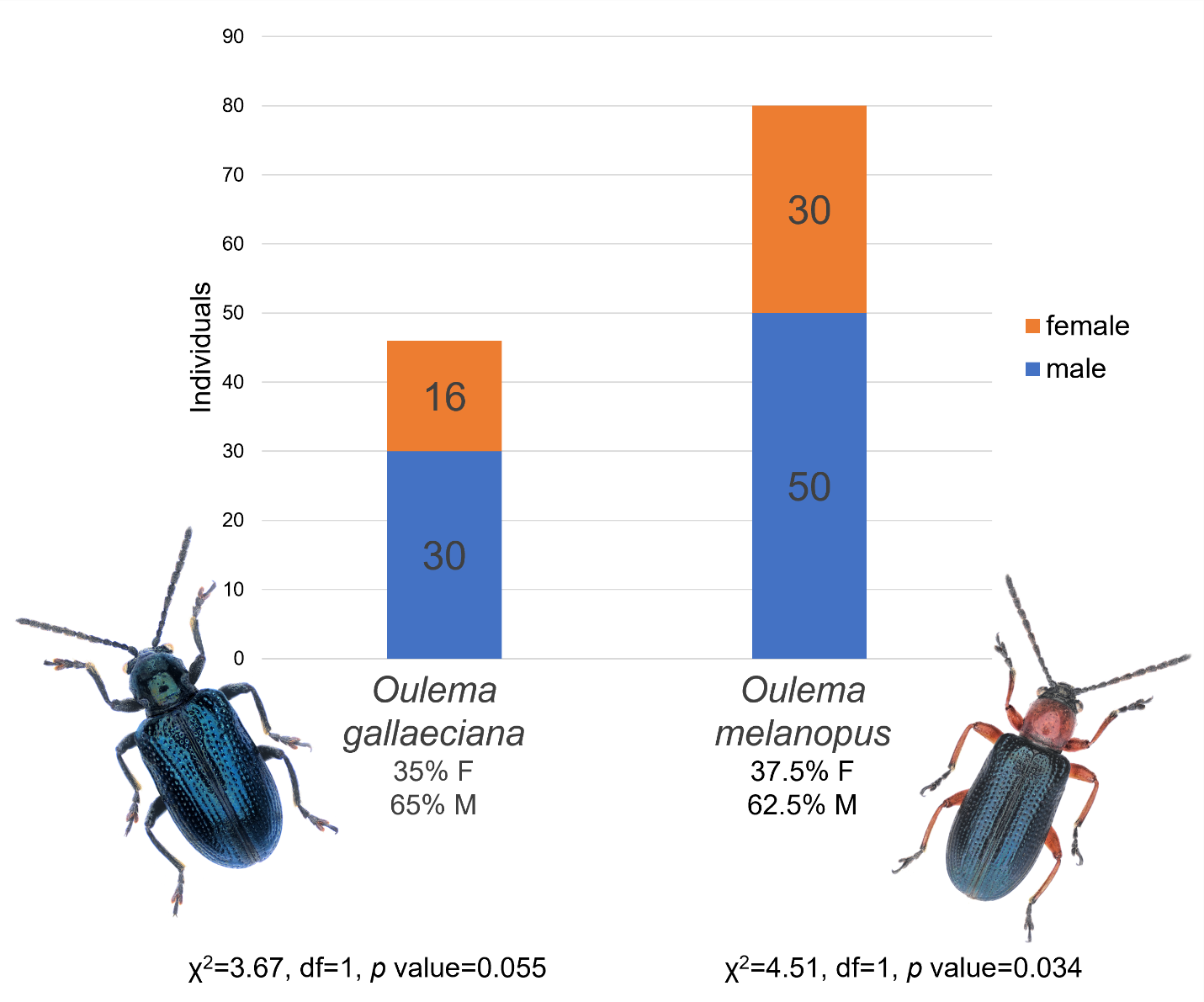


**Figure S3: Sex ratio of field caught *Oulema gallaeciana* and *Oulema melanopus***

Sex ratio was determined by dissecting field-caught adults. For statistical analysis, the one-sample proportions test with continuity correction was used. Beetle pictures from Wikimedia Commons (U. Schmidt).

**
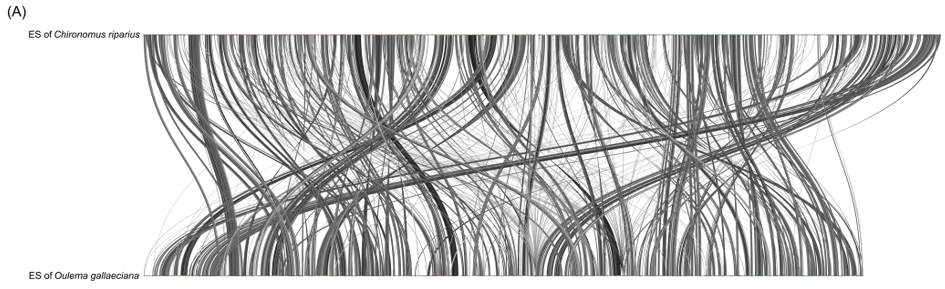
**

**
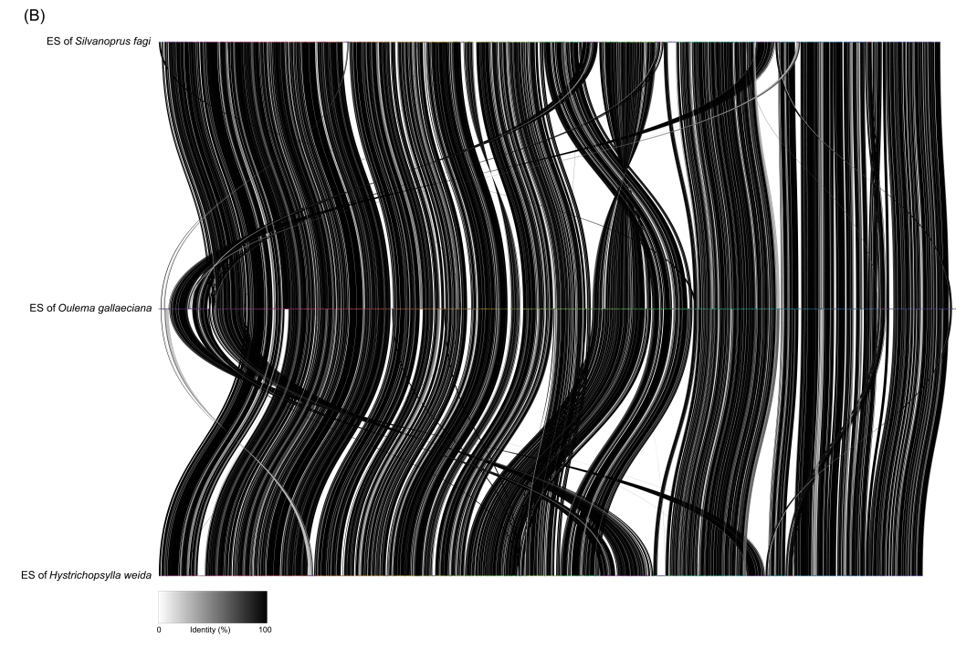
**

**Figure S4: Gene synteny of different *Symbiodolus* chromosomes.**

(A) Comparison of chromosomes of the clade 2 representative *Symbiodolus* endosymbionts (ES) of *Chironomus riparius* with the chromosome of clade 3 representative *Symbiodolus clandestinus* ES of *Oulema gallaeciana*. (B) Comparison of chromosomes of clade 3 symbionts, i.e., *S*. *clandestinus* ES of *Silvanoprus fagi*, *Oulema gallaeciana*, and *Hystrichopsylla weida*. Different shades of grey connecting homologous sequences of different chromosomes indicate the percentage of identity.

**Table S1**: Diagnostic and quantitative PCR primers, 16S microbial community amplicon primers, and FISH probes used in this study. All primers and probes were designed to bind in the 16S rRNA gene region.

| **Name** | **Sequence 5´-3´** | **fwd./rev.** | **Label** | **Length** | **Target** | **Use** | **Reference** |
| --- | --- | --- | --- | --- | --- | --- | --- |
| fD1 | AGAGTTTGATCCTGGCTCAG | fwd | - | 20 | general eubacterial | PCR | ^1^ |
| rP2 | ACGGCTACCTTGTTACGACTT | rev | - | 21 | general eubacterial | PCR | ^1^ |
| Chiro_ripa_ES_fwd01 | CCACTTGCCATTAGAGGAACC | fwd | - | 21 | *Symbiodolus* in *Chironomus riparius* | diagnostic PCR | This study |
| Chiro_ripa_ES_rev01 | CTACGCATTTCACCGCTAC | rev | - | 19 | *Symbiodolus* in *Chironomus riparius* | diagnostic PCR | This study |
| Ogalla_fwd01 | TAGGGCGTGCAGCTAAGAC | fwd | - | 19 | *Symbiodolus* in *Oulema* | qPCR | This study |
| Ogalla_rev02 | GCTTTAAGCCGCAATCTCC | rev | - | 19 | *Symbiodolus* in *Oulema* | qPCR | This study |
| 341f | CCTACGGGNGGCWGCAG | fwd | - | 17 | general eubacterial | Microbial community | ^2^ |
| 806bR | GGACTACNVGGGTWTCTAAT | rev | - | 20 | general eubacterial | Microbial community | ^3,4^ |
|  |  |  |  |  |  |  |  |
| EUB338 | GCTGCCTCCCGTAGGAGT | rev | cy7  Rhoda-mine Green | 18 | general eubacterial | FISH | ^5^ |
| EUB784 | TGGACTACCAGGGTATCTAATCC | rev | cy7 | 23 | general eubacterial | FISH | ^6^ |
| Thros_Phorni_Entero_cy3 | TCTTAGCTGCACGCCCTA | rev | cy3 | 18 | *Symbiodolus* in clade 3 taxa | FISH | This study |
| Chiro_ripa01_ES_cy3 | GCTCACCAGTCTTAAATGCCATTCC | rev | cy3 | 25 | *Symbiodolus* in *Chironomus riparius* | FISH | This study |
| Chiro_ripa02_ES_cy3 | GGTTCCTCTAATGGCAAGTGG | rev | cy3 | 21 | *Symbiodolus* in *Chironomus riparius* | FISH | This study |
| Wolb_W2-Cy5 | CTTCTGTGAGTACCGTCATTATC | rev | cy5 | 23 | *Wolbachia* | FISH | ^7^ |
| Wolb_Wol3_Cy5 | TCCTCTATCCTCTTTCAATC | rev | cy5 | 20 | *Wolbachia* | FISH | ^8^ |

**Table S2**: List of species in which *Symbiodolus* was identified. Source gives the country in which the host taxa were collected or the GenBank source from NCBI.

| **Host** | | | | **Source** |
| --- | --- | --- | --- | --- |
| **Order** | **Family** | **Genus** | **Species** | **Location or NCBI** |
| Coleoptera | Chrysomelidae | *Oulema* | *gallaeciana* | Germany |
| Coleoptera | Chrysomelidae | *Oulema* | *melanopus* | Germany |
| Coleoptera | Curculionidae | *Anthonomus* | *rectirostris* | Germany |
| Coleoptera | Curculionidae | *Dendroctonus* | *ponderosae* | SRR6279166 |
| Coleoptera | Curculionoidae | *Irenimus* | *aequalis* | KJ494864 |
| Coleoptera | Curculionidae | *Nedyus* | *quadrimaculatus* | Germany |
| Coleoptera | Curculionidae | *Phyllobius* | *maculicornis* | Germany |
| Coleoptera | Curculionidae | *Phyllobius* | *roboretanus* | Germany |
| Coleoptera | Curculionidae | *Polydrusus* | *formosus* | Germany |
| Coleoptera | Eucnemidae | *Microrhagus* | sp. ZM-2022 | SRR20721972 |
| Coleoptera | Nitidulidae | *Meligethes* | *atratus* | SRR16308437 |
| Coleoptera | Silvanidae | *Silvanoprus* | *fagi* | Germany |
| Coleoptera | Staphylinidae | *Deinopsis* | *erosa* | SRR5176562 |
| Coleoptera | Staphylinidae | *Gymnusa* | *konopackii* | SRR13086450 |
| Coleoptera | Staphylinidae | *Atheta* | *vestita* | SRR13086439 |
| Coleoptera | Throscidae | *Pactopus* | *hornii* | Canada |
| Diptera | Anthomyiidae | *Leucophora* | *obtusa* | SRR11469949 |
| Diptera | Chironomidae | *Chironomus* | *riparius* | OU907312 / Lab reared |
| Diptera | Chironomidae | *Clunio* | *marinus* | ERR845300 |
| Ephemeroptera | Caenidae | *Caenis* | *horaria* | SRR12518764 |
| Hemiptera | Lophopidae | *Paracorethrura* | *iocnemis* | OQ099617 |
| Lepidoptera | Hesperiidae | *Alychna* | *victa* | SRR7174557 |
| Siphonaptera | Hystrichopsyllidae | *Hystrichopsylla* | *weida* | SRR18186810 |

# References

1. Weisburg, W. G., Barns, S. M., Pelletier, D. A. & Lane, D. J. 16S ribosomal DNA amplification for phylogenetic study. *J. Bacteriol.* **173**, 697–703 (1991).

2. Herlemann, D. P. *et al.* Transitions in bacterial communities along the 2000 km salinity gradient of the Baltic Sea. *ISME J.* **5**, 1571–1579 (2011).

3. Caporaso, J. G. *et al.* Ultra-high-throughput microbial community analysis on the Illumina HiSeq and MiSeq platforms. *ISME J.* **6**, 1621–1624 (2012).

4. Caporaso, J. G. *et al.* Global patterns of 16S rRNA diversity at a depth of millions of sequences per sample. *Proc. Natl. Acad. Sci. U.S.A.* **108 Suppl 1**, 4516–4522 (2011).

5. Amann, R. I. *et al.* Combination of 16S rRNA-targeted oligonucleotide probes with flow cytometry for analyzing mixed microbial populations. *Environ. Microbiol.* **56**, 1919–1925 (1990).

6. Pakwan, C. *et al.* Bacterial communities associated with the ectoparasitic mites Varroa destructor and Tropilaelaps mercedesae of the honey bee (Apis mellifera). *FEMS Microbiol. Ecol.* **94**, (2018).

7. Heddi, A., Grenier, A. M., Khatchadourian, C., Charles, H. & Nardon, P. Four intracellular genomes direct weevil biology: nuclear, mitochondrial, principal endosymbiont, and Wolbachia. *Proc. Natl. Acad. Sci. U.S.A.* **96**, 6814–6819 (1999).

8. Sanguin, H. *et al.* Development and validation of a prototype 16S rRNA-based taxonomic microarray for Alphaproteobacteria. *Environ. Microbiol.* **8**, 289–307 (2006).
